# Predictive model for the preparedness level of the family caregiver

**DOI:** 10.1111/ijn.13057

**Published:** 2022-04-06

**Authors:** Belen Gutierrez‐Baena, Carmen Romero‐Grimaldi

**Affiliations:** ^1^ Nursing Faculty Salus Infirmorum University of Cadiz Cadiz Spain; ^2^ Hospital Viamed Bahia de Cádiz Chiclana de la Frontera Cadiz Spain; ^3^ Centro de Investigación Biomédica en Red de Salud Mental (CIBERSAM) Instituto de Salud Carlos III Madrid Spain

## Abstract

**Background:**

Many caregivers are insufficiently prepared, and little is known about measures that can be employed to enhance their preparedness.

**Aim:**

The aim of this study was to explore the factors associated with caregiver preparedness and establish a predictive model including the relationship between preparedness, burden, resilience and anxiety.

**Design:**

A cross‐sectional design was used.

**Methods:**

The sample included 172 family caregivers who were selected from one private hospital and daytime nursing centres. Caregivers were recruited from 2018 to 2019; they completed assessments for caregiver preparedness, anxiety, resilience and burden. A multiple linear regression analysis was performed to identify the factors associated with preparedness.

**Results:**

Preparedness was significantly associated with high levels of resilience and a low level of burden, while it was not associated with anxiety. Caregivers' gender, experience and cohabitation status were the main predictors. Resilience is an explanatory factor for caregiver preparedness in the predictive model.

**Conclusion:**

The demographic variables related to preparedness can be used to guide efforts to meet the needs of vulnerable caregivers. A caregiver's preparedness depends on their level of burden and resilience. Nursing interventions focused on these aspects could make the caregiver's role easier and improve the quality of care provided.

## Summary statement

What is already known about this topic?

- Many caregivers are inadequately prepared for this role, and ensuring adequate preparedness is important for family caregivers.
- Preparedness and factors predictive of this in Spanish family caregivers have not been well‐documented.
- Resilience, burden and anxiety affect caregivers, but the degree to which they influence caregivers' preparation levels is unknown.

What is the contribution of this paper?

- Specific factors influence the preparedness of family caregivers. Men and caregivers who do not cohabit in the same household with the care‐dependent person may present greater deficits in preparedness. Moreover, poor health and lack of experience in caring can result in inadequate caregiving performance.
- Readiness, resilience and burden are significantly related, and the best predictor of family caregivers' preparedness is resilience.
- Resilience leads to greater preparedness in caregivers, and better‐prepared caregivers experience a lighter burden.

What are the implications of this paper?

- Knowing the factors associated with preparedness in advance will allow health‐care professionals to prevent deficits in vulnerable family caregivers.
- Resilience is a decisive factor which prevents negative consequences such as low preparedness. A resilient coping style can diminish the burden and promote successful adaptation in caregivers.

## 1 INTRODUCTION

The phenomenon of care dependency is becoming increasingly important, especially within families. In many countries, most dependent people are cared for at home; thus, family caregivers play an essential role in caring for patients. Consequently, caregivers must often face challenges and deal with health issues and psychological, financial and social problems. Family caregivers may experience a physical, psychological, social, functional and spiritual burden (Ferrell & Mazanec, 2009). Increasing caregiver preparedness significantly improves the health outcomes of patients and quality of life of caregivers (Araújo et al., 2015). Conversely, a lack of preparedness can impact negatively on the structure, functioning and relationships of a family, thus affecting its ability to respond effectively to care‐related demands (Canga et al., 2011; Juntunen et al., 2018).

### 1.1 Background

Family caregivers are not professionally engaged in caring for other people. They are not paid for their time and play a key role in delivering care and support services to patients suffering from a chronic illness or disability (Family Caregiver Alliance, 2006). Family caregivers often execute numerous tasks that involve providing physical and emotional care while providing existential support and home maintenance (Hudson & Payne, 2011).

Caregiver preparedness has been defined as the perceived level of preparation of caregivers to cope with meeting the physical and emotional needs of patients, including the caregiver's perception of their ability to coordinate services for the care recipient, handle emergency situations and deal with the stress of their role (Archbold et al., 1990). Family caregivers do not normally receive training. Insufficient preparation can cause significant anxiety or burdens for the family caregiver (Araújo et al., 2015; Pucciarelli et al., 2014) and can lead to hospital readmission and early institutionalization for the care‐dependent person (Liu et al., 2020). Moreover, experienced family caregivers often report that they are underprepared for the role, lack adequate training, require information and psychosocial support and are dissatisfied with the available technical and emotional support (Funk et al., 2010; Shyu et al., 2010; Wang et al., 2018). Increasing family caregiver training reduces worry, depression, burden and anxiety (Karabulutlu et al., 2021; Lester et al., 2020; Petruzzo et al., 2019); conversely, unprepared family caregivers experience a low quality of life (Rochmawati & Prawitasari, 2021). Therefore, we must develop greater awareness of caregivers' needs and understand their training requirements and provide psychological counselling to improve their skills holistically.

Resilience, or the ability to recover when faced with stressful situations (Sihvola et al., 2022), is also linked to the caregiver's role. In fact, resilience increases care readiness (Lester et al., 2020; Zale et al., 2018), and having the ability to adequately cope with issues decreases a caregiver's burden and anxiety (Li et al., 2018). Therefore, it is understood that resilience, burden and anxiety are related; however, we do not know the exact connection between them and caregivers' preparedness.

Socio‐demographic factors, such as age, gender, relationship to the person and whether the caregiver is cohabiting with the patient, and perceived social support influence caregiver preparedness (Karabulutlu et al., 2021; Liu et al., 2020; Winterling et al., 2021). If the demographic characteristics of caregivers influence their preparedness, they can be addressed. Knowledge of the characteristics associated with quality of care is essential for recognizing caregivers with little preparation and avoiding the negative impact of unprepared caregivers.

Interventions aimed at increasing caregiver preparedness are important for both patients and their caregivers (Bilgin & Ozdemir, 2021). The transition from hospital to home represents a vulnerable moment for care‐dependent people, as their caregivers must learn to face new challenges. Uncertainty about how to provide care or a lack of support or information can increase caregiver stress and lead caregivers to compromise their own well‐being and effectiveness (Kuzmik et al., 2021). It is common for caregivers to request information from nurses. Nursing interventions are known to be effective, and information provided by nurses can have significant positive effects on family caregivers' feelings of readiness (Alvariza et al., 2020; Henriksson, Årestedt, et al., 2012; Holm et al., 2016). Therefore, an updated model focusing on caregiver preparedness is required to predict the preparedness of family caregivers, anticipate their need for training activities and avoid negative caregiving effects.

The aim of this study was to explore the factors associated with caregiver preparedness and establish a predictive model including the relationship between preparedness, burden, resilience and anxiety.

## 2 METHODS

### 2.1 Design

This descriptive, correlational, cross‐sectional study was conducted in Cadiz in southern Spain.

### 2.2 Sample

For convenience, non‐probabilistic sampling was conducted. The sample included 172 principal caregivers who were selected from one private hospital and daytime nursing centres. Caregivers living with the care‐dependent person and ones who lived alone were included. All the participants spent many hours with their relatives every day of the week.

The following inclusion criteria were used for the caregivers: They had to be related to the care‐dependent person, over 18 years old, of Spanish nationality, lacking in cognitive impairments, willing to provide written notice of informed consent and they could not be health‐care professionals as caregivers. Patients' dependency was assessed for example as moderate, severe or total dependency according to the Barthel index (BI; Cid‐Ruzafa & Damián‐Moreno, 1997). The BI is one of the most widely used tools to evaluate an individual's ability to perform daily activities. Each task was assigned a score of 0, 5, 10 or 15 points. The sum of the scores determines the level of dependency, which varies from 0 to 100. Care dependency was classified as follows: from 0 to 15 points (total), 20 to 35 points (severe), 40 to 55 points (moderate), 60 to 95 points (light) and 100 points (independent) (Minosso et al., 2010).

### 2.3 Data collection

The data were collected between October 2018 and June 2019 by two researchers. Four instruments were used, and the socio‐demographic data of the patients/caregivers were collected using a questionnaire. The researchers verified beforehand that the procedure was not too onerous and that the surveys could be answered in 25–30 min, because they were short instruments. The data were collected on paper forms in collaborating centres. The caregivers recruited in the hospital completed the surveys during their stay, whereas the family caregivers recruited from daytime nursing centres completed them at home. In both cases, the surveys were collected by the researchers on a scheduled deadline.

The Caregiver Preparedness Scale (CPS) was originally developed in the United States and is used to assess caregivers' readiness (Archbold et al., 1990). The scale includes eight items, each rated on a 5‐point Likert‐type scale ranging from ‘not at all prepared’ (0) to ‘very well‐prepared’ (4). A total score from 0 to 32 was calculated by adding the responses for all the items, with a higher score indicating a greater level of preparedness. For this study, the Spanish version of the CPS (S‐CPS) was used, which has demonstrated supportive fit indices at Confirmatory Factor Analysis (CFA; e.g. Comparative Fit Index [CFI], 0.92) and reliability (Cronbach's *α* = .89) among family caregivers (Gutierrez‐Baena & Romero‐Grimaldi, 2021). A form on which the participants indicated which aspect(s) of care they needed more training in was also provided.

The 10‐item Connor–Davidson Resilience Scale (CD‐RISC10) has shown supportive fit indices at CFA (e.g. CFI, 0.94) and reliability (Cronbach's *α* = 0.89) (Connor & Davidson, 2003). Each item is rated on a 5‐point Likert‐type scale ranging from ‘never’ (0) to ‘almost always’ (4), with a total score from 0 to 40. Higher scores indicate greater levels of resilience. For this study, we utilized an abbreviated Spanish version of the scale (Notario‐Pacheco et al., 2011).

The State–Trait Anxiety Inventory (STAI), an instrument for measuring anxiety that consists of 40 items divided into two scales of 20 items, was also used. Each item is scored between 0 (*almost never*) and 3 (*almost always*). The total score ranges from 0 to 120. The higher the participant's score, the higher their anxiety level. This instrument demonstrated good reliability values in its original English version and in its Spanish version (Cronbach's *α* ≥ .90), which was used for this study (Spielberger et al., 1971).

The Zarit Burden Interview (ZBI) measures a caregiver's burden using 22 items and includes a 5‐point Likert‐type scale, with each item scored between 0 (*never*) and 4 (*usually*). The total, calculated by adding all the responses, ranges from 0 to 88, with a higher score indicating a greater burden. The ZBI is a widely used and validated test for various populations with a good index of validity (CFA; e.g. CFI, 0.96) and reliability (Cronbach's *α* ≥ .91) (Vázquez et al., 2019). The Spanish version was used in this study (Martín et al., 1996).

### 2.4 Ethical considerations

The District Health Research Ethics Committee approved the study's procedures. All the participants were informed of the purpose of the study and were assured that their data would be kept confidential; they thus signed an informed consent form before participating.

### 2.5 Data analysis

The data were analysed using SPSS version 21.0. The level of statistical significance was set at *P* < .05. Two of the researchers verified the data during data entry to ensure accuracy. A descriptive analysis of the socio‐demographic and main variables was conducted to obtain the means and standard deviations (SDs) for the continuous variables and the frequencies and percentages for the categorical variables. Groups based on readiness were established following the recommendations of Yuguero et al. (2017). The S‐CPS scores were categorized into tertiles, which were identified as ‘low’, ‘average’ and ‘high’. Because the study's sample did not fit a normal distribution curve, contrast and non‐parametric tests were used for the data analysis, and the correlations between the study's variables were calculated using Spearman's Rho test. However, caregivers' characteristics in relation to preparedness levels were analysed using the Mann–Whitney *U* and Kruskal–Wallis tests. Post hoc tests were performed.

A multiple linear regression analysis was performed to explore the factors related to caregiver preparedness and identify the proportion of variance explained by these variables. The factors that were significant in the univariate and correlation analyses were entered in the model as the independent variables for analysis with preparedness as the dependent variable. First, all the significant variables were entered in the model simultaneously (forced entry method). This full model was followed by stepwise backward elimination to determine whether each variable remained significant after non‐significant covariates were excluded. The assumptions for multiple regressions were checked using residual analysis. The assumptions of normality, linearity and homoscedasticity were generally met. No multicollinearity problems were detected across the independent variables; the mean level of the variance inflation factor (VIF) was within the allowed limits (Savin & White, 1977).

## 3 RESULTS

### 3.1 Socio‐demographic characteristics of family caregivers and care‐dependent patients and caregiving‐related variables

One hundred and seventy‐two of the 300 potential participants returned the surveys for an overall response rate of 57.3%. Most of the caregivers were middle‐aged (66%), married (75%) and female (79%). Nearly half were the child (52%) of the patient, with predominant education levels related to primary school (33%) or secondary school (38%). Slightly more than half the caregivers were in good health (55%) (Table 1).

**TABLE 1:** Characteristics of the participants

Variables	*n*	%
Gender		
Male	36	21
Female	136	79
Age range, years		
<45	19	11
45 54	44	26
55 64	69	40
65 75	24	14
>75	16	9
Relationship with care‐dependent patient		
Parent	30	17
Son/daughter	90	52
Brother/sister	10	6
Spouse	28	16
Other (uncle, nephew, grandson, son‐in‐law, daughter‐in‐law)	14	8
Marital status		
Married	129	75
Single	26	15
Widow	9	5
Divorced	4	2
Partnered	4	2
Education level		
No studies	17	10
Primary studies	56	33
Secondary studies	66	38
University studies	33	19
Employment status		
Employee	64	37
Retired	26	15
Unemployed	20	12
Homemaker	62	36
Monthly family income, euros		
<501	14	8
501–1000	65	38
>1000	91	53
Caregiver health		
Good/very good	94	55
Average	71	41
Bad/very bad	7	4
Children		
Yes	49	29
No	123	72

### 3.2 Levels of anxiety, resilience, burden and preparedness among family caregivers and two‐way interactions

Most of the care‐dependent patients were female (67%) with severe/total (78%) dependency. Most (63%) of the caregivers lived with the care‐dependent patient, and approximately half of the caregivers spent more than 14 h a day (45%) providing care. Regarding those who had provided care continuously, 35% had provided care for three to 5 years, and 25% had done so for more than 10 years. Finally, we measured the health of those receiving care while considering their number of hospitalisations in the previous year, which was ≤2 in 86% of the cases (Table 2).

**TABLE 2:** Characteristics of the care‐dependent patient and care situation

Variables	*n*	%
Gender		
Male	57	33
Female	115	67
Barthel's index score as dependency		
Total	71	41
Severe	63	37
Moderate	36	21
Slight	2	1
Cohabitation with the care‐dependent patient		
Yes	109	63
No	63	37
Hours per day dedicated to caregiving		
2–8	52	30
9–13	42	24
≥14	78	45
Time spent as a caregiver, years		
0–2	35	20
3–5	61	35
6–10	33	19
>10	43	25
Number of hospitalizations per year		
0–2	147	86
3–5	14	8
>5	11	6

The average scores obtained on the scales and the results for the two‐way interaction variables are presented in Table 3. Caregiver preparedness was directly related to caregiver resilience and inversely related to caregiver burden. We found no statistically significant associations between preparedness and anxiety. Additionally, we identified a significant relationship between burden and anxiety (*r* = .320; *P* < .001) and resilience (*r* = −.280; *P* < .001).

**TABLE 3:** Caregiver preparedness among family caregivers and correlation with anxiety, resilience, and burden

Variable (scale)	Correlation coefficients with preparedness^a^		Mean (SD)	Range	
	*r*	*P* value			
Preparedness (S‐CPS)	1		20.07 (6.4)	0–32	
Anxiety (STAI)	.051	.510	51.71 (9.4)	31–79	
Resilience (CD‐RISC10)	**.533**	**<.001**	26.95 (7.6)	3–40	
Burden (ZBI)	**−.296**	**<.001**	34.06 (14.8)	1–76	
S‐CPS score	S‐CPS score	Preparedness groups			*n*%
<19	Low preparedness		12.84 (4.9)	56	32
19–23	Average preparedness		20.95 (1.3)	62	36
≥24	High preparedness		26.56 (2.5)	54	31

Abbreviations: CD‐RISC10, Connor–Davidson Resilience Scale; S‐CPS, Spanish Caregiver Preparedness Scale; SD, standard deviation; STAI, State–Trait Anxiety Inventory; ZBI, Zarit Burden Interview.

^a^ Spearmans Rho correlation coefficient.

The preparedness measured by the S‐CPS in our caregiver sample was 20.07 (range 0–32). The mean score per item was 2.51 (range 0–4). To help us distinguish the participants who were well‐prepared from those who were not, we divided the sample into preparedness‐based groups using the tertile method. More than 67% of the sample demonstrated low or moderate preparedness, whereas 54 of the 172 participants had high preparedness (Table 3). We also asked the participants about the areas of care in which they felt they needed to improve. The most frequently cited area was coping (44%). Some additional areas for improvement included technical skills (23%), information on social services and the health‐care system (36%) and medication and skincare (33% and 28%, respectively).

### 3.3 Factors associated with preparedness

The univariate analysis demonstrated that there was a significant relationship between preparedness and caregivers' sociodemographic characteristics and the variables related to the caregiving situation, including gender, relationship (*P* < .001), age, duration of care, health of the caregiver (*P* < .01), marital status and cohabitation status (*P* < .05) (Table 4). The post hoc analysis indicated that women (*P* < .001) who had been caregiving for more than 6 years (*P* < .01) and who lived with the care‐dependent person (*P* < .05) had greater preparedness. Furthermore, caregivers over 75 years old and with poor health represented the group with the least preparedness ( $\bar{x}=14.06$; SD = 7.8, $\bar{x}=10.00$; SD = 9.8, respectively). We found no significant relationship between preparedness and other factors, such as the caregiver's education level and employment status and the presence of children.

**TABLE 4:** Univariate analysis of caregiver preparedness

Variable	Statistical value	*P* value
Gender (*z*)^a^	1.537	.001***
Age (*ϰ* ^2^)^b^	13.911	.008**
Education level (*ϰ* ^2^)^b^	2.593	.459
Marital status (*ϰ* ^2^)^b^	13.012	.011*
Relationship with patient (*ϰ* ^2^)^b^	28.349	<.001***
Employment status (*ϰ* ^2^)^b^	4.001	.261
Length of time as a caregiver (years) (*ϰ* ^2^)^b^	13.350	.004**
Hours per day dedicated to caregiving (*ϰ* ^2^)^b^	3.464	.177
Cohabitation status (*z*)^a^	2.659	.014*
Presence of children (*z*)^a^	2.629	.218
Monthly income (*ϰ* ^2^)^b^	.025	.987
Caregivers health status (*ϰ* ^2^)^b^	11.262	.004**

^a^ Mann–Whitney *U* test.

^b^ Kruskal–Wallis test.

* *P* < .05.

** *P* < .01.

*** *P* < .001.

A multiple regression analysis was used to examine the factors related to caregiver preparedness using caregiver preparedness as the dependent variable and the significant factors in the univariate and Spearman's Rho correlation analysis as independent variables. The caregiver's gender (*P* < .001), duration of care (*P* < .01), cohabitation status (*P* < .05), resilience (*P* < .001) and burden (*P* < .05) had a significant relationship with caregiver preparedness. Resilience was the most prominent factor among the variables. Forty‐four per cent of the variance in caregiver preparedness was explained by the above‐mentioned factors (Table 5).

**TABLE 5:** Factors related to caregiver preparedness in the multiple regression analysis

Variables	*B*	Beta	*t*	*P* value
Gender (female)	3.389	.216	3.416	.001***
Caregivers age	−.266	−.045	−.647	.519
Relationship	.171	.034	.538	.591
Time as a caregiver (years)	1.158	.195	3.143	.002**
Cohabitation status (yes)	−1.983	−.150	−2.367	.019*
Caregivers health	−.673	−.061	−.863	.389
Resilience	.362	.432	6.494	<.001***
Burden	−.070	−.162	−2.500	.013*

*Note*: Beta = standardized coefficient; *R* = multiple correlation coefficient; *R* ^2^ = adjusted coefficient of determination; *F* = Fishers *F* test. *R* = .664, *R* ^2^ = .441, *F* = 16.053, *P* < .01.

* *P* < .05.

** *P* < .01.

*** *P* < .001.

The results of the hierarchical multiple regression analysis are presented in Table 6. To construct a predictive model for caregiver preparedness, only the factors that were significantly associated with it in the bivariate analysis were selected and entered in the model as predictive variables. The various models represent the direct effects of the control and predictor variables. Regarding the results for caregiver preparedness, the hierarchical entry of the predictor variables resulted in statistically significant increases in *R* ^2^, with the model that encompasses five variables (resilience, burden, duration of care, gender and cohabitation status) identified as the one with the highest adjusted coefficient of determination.

**TABLE 6:** Hierarchy of multiple regression analysis of caregiver preparedness

Model	Variables included	*R* ^2^ adjusted (%)	*P* value
1	Resilience	30.1	<.001
2	Resilience and length of time as a caregiver (years)	35.7	<.001
3	Resilience, length of time as a caregiver (years) and gender	37.9	<.001
4	Resilience, length of time as a caregiver (years), gender and burden	40.1	<.001
5	Resilience, length of time as a caregiver (years), gender, burden and cohabitation status	41.7	<.001

*Note*: *R* ^2^ adjusted = adjusted coefficient of determination.

**FIGURE 1:**
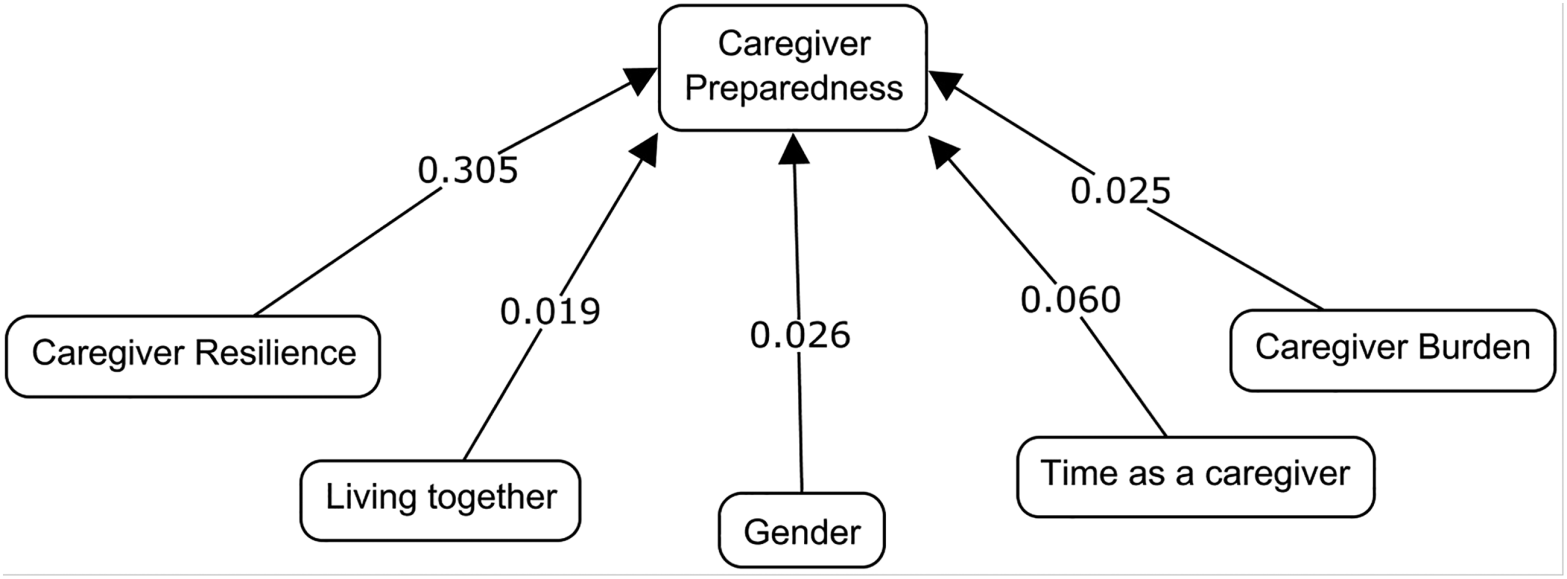
Factors involved in caregiver preparedness. *Note*: The data show the proportion of variability in the model

Table 7 presents the final multiple regression model used to predict family caregivers' preparedness. The multiple regression model explained 43.4% of the caregiver preparedness variance (*F* = 25.487, *P* < .001). Resilience, duration of care (more years), gender (female), burden and cohabitation status (yes) explained 30.5%, 6%, 2.6%, 2.5% and 1.9% of the variance in caregiver preparedness, respectively (Figure 1). Specifically, the greater the resilience and the caring experience, the greater the preparedness of the caregiver. On the contrary, burden decreased preparation. Women caregivers had higher preparedness than men, and so did caregivers who cohabit with the care‐dependent patient. Following the residual analysis, the proposed regression model complied with the assumptions of autocorrelation (Durbin Watson = 2.064), collinearity (VIF = between 1.04 and 1.16), linearity, normality and homoscedasticity.

**TABLE 7:** Final multiple linear regression analysis model of variables related to family caregivers preparedness

Independent variables	*B*	Standard error	Beta	*t*	*P* values	95% CI	VIF
Constant	6.124	2.616		2.341	.020	.96; 11.29	
Resilience	.384	.053	.457	7.268	<.001	.28; .49	1.162
Duration of care	1.101	.358	.186	3.072	.002	.39; 1.81	1.071
Gender (female)	3.397	.975	.217	3.485	.001	1.47; 5.32	1.133
Burden	−.078	.027	−.181	−2.880	.005	−.13; −.03	1.157
Cohabitation status (yes)	−1.867	.787	−.141	−2.372	.019	−3.42; −.31	1.036
Model statistics *R* = .659, *R* ^2^ = .434, adjusted *R* ^2^ = .417, *P* < .001, *F* = 25.487.							

*Note*: *B* = regression coefficients; Beta = standardized regression coefficients; *R* = multiple correlation coefficient; *R* ^2^ = adjusted coefficient of determination; *F* = Fishers F test.

Abbreviations: CI, confidence interval; VIF, variance inflation factor.

## 4 DISCUSSION

The aim of this study was to evaluate the preparedness of a sample of caregivers, determine the associated factors and examine the relationship between preparedness and anxiety, resilience and burden.

The profile of family caregivers, who were predominantly married women between 45 and 64 years old with a basic education who dedicated more than 9 h a day to caregiving (Table 1), was presented. This profile mirrors those described by other researchers (Perpiñá‐Galvañ et al., 2019; Petruzzo et al., 2019; Vellone et al., 2020). The primary caregiver is often a first‐degree relative or spouse who spends many hours a day providing care. Many of the caregivers in our sample had provided care for many years and lived with the recipient (Gutierrez‐Baena & Romero‐Grimaldi, 2021; Henriksson, Andershed, et al., 2012). Caregiving often becomes a constant task that exhausts the caregiver, and such exhaustion often manifests as physical and psychological problems that can lead to more serious pathologies in caregivers (Yang et al., 2019). However, caregiving is a tremendously important task, as 34% of the elderly require care from relatives (European Commission, 2021). We obtained a mean score per item of 2.51 (range 0–4) on the preparedness scale. Other researchers have used the same instrument, which has been validated in various languages (Grant et al., 2013; Pucciarelli et al., 2014). These studies showed mean scores per item ranging from 1.80 (China) (Liu et al., 2020) to 3.73 (North America) (Fujinami et al., 2015). In Latin America, the ability of caregivers to provide care has been measured using the Care Ability Inventory (CAI), which showed that more than 70% of the population needed to strengthen its caregiving abilities (Benítez & Carreño Moreno, 2015) and demonstrated that populations in various geographical settings can have different levels of caregiving preparedness.

When separating the sample into groups based on caregiver preparedness (Schumacher et al., 2007), approximately 70% of the sample demonstrated low or moderate preparedness. Thus, we identified the groups with moderate and low preparedness, among which specific interventions could be conducted to improve various aspects of caregiving.

Family caregivers often feel they are poorly informed, inadequately trained and dissatisfied with the type and quality of technical and emotional support available (Shyu et al., 2010). The caregivers included in our study demanded more training on how to obtain help and information from the health‐care and social services system, information on medications and knowledge of other topics, but guidelines for coping with the demands of caregiving were most desired. Coping strategies can mitigate depressive symptoms and reduce both caregivers' anxiety and burden (Monteiro et al., 2018). Further coping strategies reduce psychological distress and improve caregivers' well‐being (Corallo et al., 2018).

Our results indicate that caregiver preparedness is inversely related to the burden (Table 3). Scherbring (2002) concluded that such feelings of burden decrease caregivers' quality of life and can reduce their preparedness. Additionally, feelings of burden are related to depression and uncertainty (Liu et al., 2020).

Low caregiver preparedness has been associated with greater anxiety and depression in the caregivers of patients with cancer (Dionne‐Odom et al., 2017). Conversely, in our study, preparedness was unrelated to anxiety (Table 3). This finding was similar to those of studies in which caregiver preparedness and anxiety were not found to be significantly related (Hendrix et al., 2016; Petruzzo et al., 2019).

We have noted that women and men experience significant differences in their feelings of caregiver preparedness. Our results and those of other studies indicated that women felt more prepared for caregiving than men, although our sample of male caregivers was small, and the male sample size should be increased in future studies to reach more definitive conclusions. In addition, family caregivers cohabiting with the recipient of care felt more prepared than those who did not cohabitate (Henriksson & Årestedt, 2013).

Surprisingly, higher educational levels among caregivers did not imply better preparation for caregiving. These results support the findings of Shyu et al. (2008), who reported that educational level was not a factor in caregiver preparedness. This may be the case because providing care requires knowledge, skills and abilities that are not taught in a formal educational context. However, caregivers often face challenges without receiving any training. Although there are training programmes and assistance plans for caregivers, such initiatives are insufficient and do not reach most of the population.

The duration of caregiving and both the health condition and age of the caregiver were associated with caregiver preparedness. Specifically, the most vulnerable group included caregivers who had been performing the role for a shorter time, had a poor health condition and were part of the oldest age group. Accordingly, our results indicated that the group that had been caregiving for more than 6 years generally felt better‐prepared. Over the years, the skills and knowledge related to caregiving accumulate, as those who have had previous caregiving experiences report greater preparedness than those who had never cared for another person (Liu et al., 2020). Similarly, a previous study established a direct relationship between the preparedness and the health condition of family caregivers (Dionne‐Odom et al., 2017).

In the multiple regression model in our study, resilience was the variable that explained a caregiver's preparedness to the greatest extent (Figure 1). A similar study with caregivers of stroke survivors explained that a caregiver's preparedness was based on their previous experience (Liu et al., 2020). This may be due to the resilience of more experienced caregivers compared with those with little or no experience. We can envision how caregiving experiences might shape someone's personality and give them the strength to face difficult situations. To summarize the results of the model, which identified resilience as the most important factor, caregivers who displayed greater preparedness also exhibited greater resilience in their roles as family caregivers (Table 3). These findings support other studies' results, which have established a direct relationship between preparedness and the ability to cope with adverse situations (Lester et al., 2020). This could explain why coping skills are one of the areas in which improvement is most desired by caregivers (own results). Furthermore, we determined that the burden felt by caregivers was significantly negatively associated with resilience; that is, the heavier the burden, the lower their resilience. This result supports those of a previous study that demonstrated that the family caregivers of cancer patients felt less burdened as their level of resilience increased (Li et al., 2018). Resilience is an individual personality trait. Caring for someone for many hours each day involves overcoming difficulties constantly. Therefore, resilience provides caregivers with a greater ability to cope with difficult situations; that is, it improves their ability to care for others, and we concluded that it is one of the strongest predictors of caregiver preparedness. However, these new results raise a new question: Can we enhance the resilience of individuals to improve their skills as caregivers?

Finally, we now understand better the factors that predict family caregivers' preparedness and have a predictive model to identify which factors are the most important. Thus, the focus must be placed on these factors when caregivers are trained by nurses, who play an important role in their education, because it has been shown that psychoeducational interventions by nurses help caregivers cope with the demands of care (Leow et al., 2015). Additional research is needed to identify the other factors affecting caregiver readiness and whether these results are applicable to caregivers of patients with diverse pathologies. Therefore, to increase caregiver preparedness, it is necessary to understand the factors limiting their progress and establish models to predict how preparedness affects family caregivers.

### 4.1 Limitations

This study had several limitations. First, we recruited a convenience sample that included only caregivers from a single region in Spain. Second, this study was cross‐sectional; thus, a temporal relationship between the variables was not established. Therefore, validating the model with covariates observed over time is recommended. Considering the above limitations, the generalizability of our results may be limited.

## 5 CONCLUSIONS

Caregivers need greater preparedness to live up to their role. On top of this, there are factors that will establish the groups of caregivers with low preparedness. Those who may present greater deficits in preparedness are men and caregivers who do not cohabit in the same household with the care‐dependent person. Moreover, a poor health condition and lack of experience in caring will result in an inadequate caregiving performance. On the other hand, we can affirm that one of most important skills for people who look after care‐dependent patients is a higher capacity for coping with difficult situations. A resilient attitude can promote adaptation and diminish the risk of burden. Further research will be needed to explore other factors related to preparedness of caregivers and to make use of these findings in developing and trialling nursing interventions with these vulnerable groups.

## CONFLICT OF INTEREST

The authors declare no conflicts of interest.

## AUTHORSHIP STATEMENT

The authors are responsible for all aspects of the work and all listed authors meet the authorship criteria.

## Data Availability

The datasets used during the current study are available from the corresponding author on reasonable request.
